# Polymyxin Combinations Combat *Escherichia coli* Harboring *mcr-1* and *bla*_NDM-5_: Preparation for a Postantibiotic Era

**DOI:** 10.1128/mBio.00540-17

**Published:** 2017-07-25

**Authors:** Zackery P. Bulman, Liang Chen, Thomas J. Walsh, Michael J. Satlin, Yuli Qian, Jürgen B. Bulitta, Charles A. Peloquin, Patricia N. Holden, Roger L. Nation, Jian Li, Barry N. Kreiswirth, Brian T. Tsuji

**Affiliations:** a Laboratory for Antimicrobial Dynamics, NYS Center of Excellence in Bioinformatics and Life Sciences, Buffalo, New York, USA; b School of Pharmacy and Pharmaceutical Sciences, University at Buffalo, Buffalo, New York, USA; c Public Health Research Institute, New Jersey Medical School, Rutgers, the State University of New Jersey, Newark, New Jersey, USA; d Weill Cornell Medical College, Cornell University, New York, New York, USA; e Center for Pharmacometrics and Systems Pharmacology, Department of Pharmaceutics, College of Pharmacy, University of Florida, Orlando, Florida, USA; f Department of Pharmacotherapy and Translational Research, University of Florida, College of Pharmacy, Gainesville, Florida, USA; g Drug Delivery, Disposition and Dynamics, Monash Institute of Pharmaceutical Sciences, Monash University, Melbourne, Australia; McMaster University

**Keywords:** *Enterobacteriaceae*, MCR-1, NDM-5, amikacin, aztreonam, carbapenem-resistant, polymyxins

## Abstract

The rapid increase of carbapenem resistance in Gram-negative bacteria has resurrected the importance of the polymyxin antibiotics. The recent discovery of plasmid-mediated polymyxin resistance (*mcr*-*1*) in carbapenem-resistant *Enterobacteriaceae* serves as an important indicator that the golden era of antibiotics is under serious threat. We assessed the bacterial killing of 15 different FDA-approved antibiotics alone and in combination with polymyxin B in time-killing experiments against *Escherichia coli* MCR1_NJ, the first reported isolate in the United States to coharbor *mcr-1* and a New Delhi metallo-β-lactamase gene (*bla*_NDM-5_). The most promising regimens were advanced to the hollow-fiber infection model (HFIM), where human pharmacokinetics for polymyxin B, aztreonam, and amikacin were simulated over 240 h. Exposure to polymyxin B monotherapy was accompanied by MCR1_NJ regrowth but not resistance amplification (polymyxin B MIC from 0 to 240 h [MIC_0h_ to MIC_240h_] of 4 mg/liter), whereas amikacin monotherapy caused regrowth and simultaneous resistance amplification (amikacin MIC_0h_ of 4 mg/liter versus MIC_240h_ of >64 mg/liter). No MCR1_NJ colonies were observed for any of the aztreonam-containing regimens after 72 h. However, HFIM cartridges for both aztreonam monotherapy and the polymyxin B-plus-aztreonam regimen were remarkably turbid, and the presence of long, filamentous MCR1_NJ cells was evident in scanning electron microscopy, suggestive of a nonreplicating persister (NRP) phenotype. In contrast, the 3-drug combination of polymyxin B, aztreonam, and amikacin provided complete eradication (>8-log_10_ CFU/ml reduction) with suppression of resistance and prevention of NRP formation. This is the first comprehensive pharmacokinetic/pharmacodynamic study to evaluate triple-drug combinations for polymyxin- and carbapenem-resistant *E. coli* coproducing MCR-1 and NDM-5 and will aid in the preparation for a so-called “postantibiotic” era.

## IMPORTANCE

A global health crisis may be on the horizon, as the golden era of antibiotics is under serious threat. We recently reported the first case in the United States of a highly resistant, *Escherichia coli* so-called “superbug” (MCR1_NJ), coharboring two of the most worrying antibiotic resistance genes, encoding mobile colistin resistance (*mcr*-*1*) and a New Delhi metallo-β-lactamase (*bla*_NDM-5_). Worryingly, the medical community is vulnerable to this emerging bacterial threat because optimal treatment strategies are undefined. Here, we report the activity of an optimized combination using simulated human doses of commercially available antibiotics against MCR1_NJ. A unique triple combination involving a cocktail of polymyxin B, aztreonam, and amikacin eradicated the MCR-1- and NDM-5-producing *E. coli*. Each antimicrobial agent administered as monotherapy or in double combinations failed to eradicate MCR1_NJ at a high inoculum. To our knowledge, this is the first study to propose 3-drug therapeutic solutions against superbugs coharboring *mcr-1* and *bla*_NDM_, seeking to prepare clinicians for future occurrences of these pathogens.

## OBSERVATION

Carbapenem-resistant *Enterobacteriaceae* (CRE) pose an urgent threat to global human health. Among the enzymes responsible for carbapenem resistance, the New Delhi metallo-β-lactamases (NDM) warrant significant attention due to their location on highly mobile genetic elements and coexistence with many other resistance determinants that have resulted in their rapid worldwide dissemination. The polymyxin antibiotics (polymyxin B and polymyxin E [colistin]) have resurged as important last-line treatment options against CRE. The World Health Organization has also reclassified the polymyxin antibiotics as being “critically important to human medicine,” highlighting the need to optimize their clinical use (1). Therefore, the recent discovery of the plasmid-mediated, mobile polymyxin resistance gene *mcr-1* poses a significant threat to the clinician’s treatment armamentarium.

MCR-1-producing *Escherichia coli* was first discovered in 2015 in China (2) and has now been detected in the United States and worldwide (3). MCR-1 is a phosphoethanolamine transferase capable of modifying the lipid A portion of lipopolysaccharide with phosphoethanolamine, thereby inhibiting the binding of polymyxins. Even more worrisome, polymyxin- and carbapenem-resistant clinical isolates have been increasingly identified, including the first MCR-1- and NDM-5-coproducing *E. coli* isolate (MCR1_NJ) in the United States, which was recently reported by our group (4). MCR1_NJ was isolated from a patient in New Jersey who had a history of prostate cancer and developed recurrent urinary tract infections. The *E. coli* urinary isolate was found to contain both *mcr-1* and *bla*_NDM-5_ genes on separate plasmids (pMCR1-NJ-IncX4 and PNDM5-NJ-IncX3, respectively), in addition to other resistance determinants for aminoglycosides, β-lactams, chloramphenicol, fluoroquinolones, rifampin, sulfonamides, and tetracycline. Worryingly, there are a growing number of similar *Enterobacteriaceae* strains with MCR-1 coexisting with NDM-1, NDM-5, and NDM-9 (5–8). Truly pan-drug-resistant (PDR) strains of *Enterobacteriaceae* harboring *bla*_NDM_ have recently been reported in the United States (9) and worldwide (10), with chromosomal mutations in *mgrB* accounting for polymyxin resistance in these isolates. Cumulatively, these findings have escalated the threat of a “postantibiotic” era; it may only be a matter of time before hospitals around the world face a large outbreak of MCR-1- and NDM-producing *Enterobacteriaceae* (11, 12).

The clinical impact of infections caused by *mcr-1-* or *bla*_NDM_-harboring bacteria is currently unknown. However, infections by CRE with non-MCR-1, chromosomally encoded polymyxin resistance mechanisms result in unacceptably high mortality rates that may be in excess of 60% (13, 14). The medical community is unprepared to treat Gram-negative pathogens harboring *mcr-1* and *bla*_NDM_ due to the lack of published *in vitro*, animal, or clinical studies evaluating the effectiveness of the limited treatment options. Therefore, it is imperative to elucidate optimized therapeutic strategies using currently available antimicrobials to therapeutically prepare for these very problematic pathogens. Here, we describe for the first time the pharmacodynamic activities of antimicrobial combinations against *E. coli* MCR1_NJ, the first reported isolate from the United States coharboring MCR-1 and NDM.

### Results.

**FIG 1:**
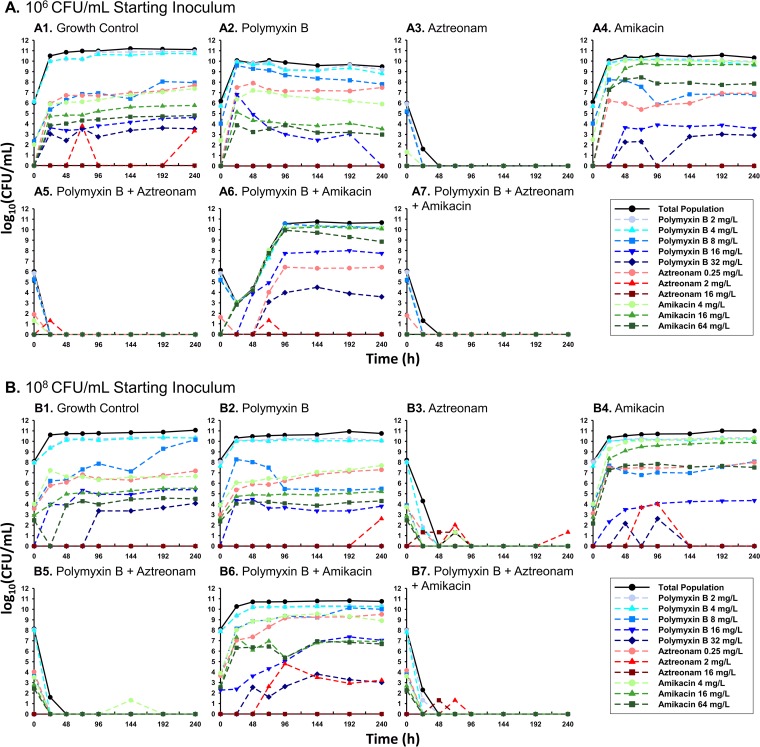
*E. coli* MCR1_NJ total population bacterial counts (black lines) and antibiotic-resistant subpopulations (colored lines) quantified during the HFIM at a starting inoculum of either ~10^6^ CFU/ml (A) or ~10^8^ CFU/ml (B). Antibiotic-resistant subpopulations, which are fractions of the respective total population, were quantified using MHA plates imbued with the specified concentrations of polymyxin B (blue lines), aztreonam (red lines), or amikacin (green lines). Humanized regimens of polymyxin B with front loading (3.33 mg/kg for 1 dose followed by 1.43 mg/kg q12h starting 12 h later), aztreonam (2 g q8h), and amikacin (15 mg/kg q24h) were simulated over 240 h.

In the dose-ranging time-kill experiments against MCR1_NJ at an ~10^6^-CFU/ml inoculum, the minimum amikacin concentration required to achieve undetectable bacterial counts at 24 h was 256 mg/liter. The addition of polymyxin B reduced the concentration of amikacin required to achieve no bacterial growth to 64 mg/liter (3.04-fold reduction in the fitted 50% effective concentration [EC_50_]). The minimum aztreonam concentration required to cause undetectable bacterial growth after 24 h was 4 mg/liter; polymyxin B reduced the aztreonam concentration requirement for undetectable growth to 1 mg/liter (3.73-fold reduction in the fitted EC_50_). At an ~10^6^-CFU/ml inoculum of *E. coli* MCR1_NJ in the hollow-fiber infection model (HFIM) (Fig. 1A1 to A7), polymyxin B and amikacin monotherapies caused maximal bacterial reductions within 6 h of 1.72 and 3.34 log_10_ CFU/ml, respectively, followed by bacterial regrowth by 24 h. A combination of polymyxin B and amikacin prolonged the time until regrowth until 96 h and resulted in maximal bacterial killing of 6.10 log_10_ CFU/ml at 30 h. Population analysis profiles (PAPs) obtained throughout exposure to amikacin monotherapy or the polymyxin B-plus-amikacin combination revealed a 3.06- and a 4.05-log_10_ CFU/ml increase of the amikacin-resistant subpopulation (64-mg/liter amikacin-imbued agar), respectively, at 240 h compared to this subpopulation in the growth control. In contrast to the amikacin resistance, MCR1_NJ polymyxin B resistance remained stable during each HFIM experiment regardless of polymyxin exposure. Aztreonam monotherapy, polymyxin B plus aztreonam, and the triple combination resulted in bacterial reductions of >6 log_10_ CFU/ml and undetectable bacterial counts that were sustained through 240 h beginning after 48 h, 24 h, and 26 h, respectively. These three antibiotic regimens also suppressed polymyxin, aztreonam, and amikacin resistance.

**FIG 2:**
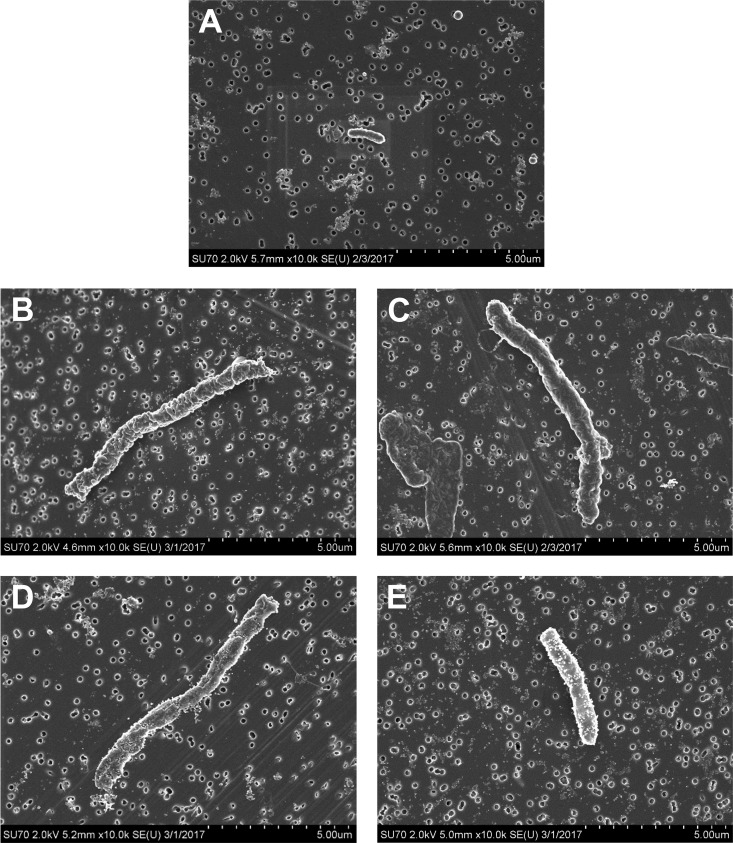
Scanning electron microscopy images of representative *E. coli* MCR1_NJ cells at a magnification of ×10,000. Images were obtained from HFIM samples at ~10^8^-CFU/ml inoculum taken prior to antibiotic exposure (A), after 24-h exposure to aztreonam (B), after 240-h exposure to aztreonam (C), after 24-h exposure to polymyxin B plus aztreonam (D), and after 240-h exposure to polymyxin B plus aztreonam (E). Bacterial samples were prepared and imaged on 0.2-μm filter paper, which is seen behind the cells for reference.

The antibiotic regimens caused similar bacterial killing and resistance patterns at the ~10^8^-CFU/ml starting inoculum of *E. coli* MCR1_NJ (Fig. 1B1 to B7); aztreonam monotherapy, polymyxin B plus aztreonam, and the triple combination all drove total bacterial counts to be undetectable (>8-log_10_ CFU/ml reductions in total bacterial counts). The most notable difference at the ~10^8^-CFU/ml starting inoculum was the marked turbidity (240-h optical density at 620 nm [OD_620_]) observed through 240 h in the aztreonam monotherapy (OD_620_ of 0.108) and polymyxin B-plus-aztreonam (OD_620_ of 0.083) HFIM cartridges, despite no observed bacterial growth, compared to the clear cultures observed only for the triple combination. Visualization of MCR1_NJ from the HFIM before and after exposure to aztreonam monotherapy or polymyxin B plus aztreonam using scanning electron microscopy (SEM) revealed an elongation of the bacterial cells from the baseline (Fig. 2). The long, filamentous *E. coli* cells from the HFIM that was exposed to aztreonam were each ~2 times wider and up to at least 6 times longer than unexposed bacterial cells. Exposure to polymyxin B plus aztreonam also induced outer membrane damage to the MCR1_NJ cells, as demonstrated by protrusions across the entire cell surface.

Treatment with amikacin or polymyxin B plus amikacin against ~10^8^ CFU/ml of MCR1_NJ caused maximal bacterial reductions after 4 h of 2.43 and 3.10 log_10_ CFU/ml, respectively, before regrowth occurred. For the 3 regimens that resulted in bacterial regrowth at the ~10^8^-CFU/ml inoculum, the polymyxin B PAPs remained relatively unchanged throughout 240 h; there was no appreciable proliferation of polymyxin-resistant subpopulations (polymyxin B MIC at 0 h [MIC_0h_] of 4 mg/liter and MIC_240h_ of 4 mg/liter). The bacterial regrowth after treatment with amikacin or polymyxin B plus amikacin was accompanied by increases of 2.99 log_10_ CFU/ml (amikacin MIC_0h_ of 4 mg/liter and MIC_240h_ of >64 mg/liter) and 2.17 log_10_ CFU/ml (amikacin MIC_0h_ of 4 mg/liter and MIC_240h_ of 32 mg/liter), respectively, in the amikacin-resistant subpopulation (64-mg/liter amikacin-imbued agar) compared to this population in the growth control. There were no aztreonam-resistant subpopulations detected after 240 h in the HFIM for any antibiotic regimen at either starting inoculum.

*E. coli* MCR1_NJ’s growth at the ~10^8^-CFU/ml inoculum when exposed to polymyxin B monotherapy closely mirrored that of the growth control. Whole-genome sequencing (WGS) after exposure to the polymyxin B front-load regimen for 24 and 240 h revealed 6 and 8 homozygous single-nucleotide polymorphisms (SNPs), respectively, (no homozygous indels) compared to the sequence of unexposed MCR1_NJ. However, none of the SNPs were detected in genes previously associated with polymyxin resistance. Further examination confirmed that there were no heterozygous variants in any of the previously identified polymyxin resistance genes. These WGS results are consistent with the stable MCR1_NJ phenotypic response to polymyxin B treatment in the HFIM (polymyxin B MIC of 4 mg/liter from 0 to 240 h). Thus, in the *mcr-1-*harboring MCR1_NJ isolate, polymyxin exposure did not potentiate polymyxin resistance through the acquisition of a new mutation.

### Discussion.

Infections caused by *Enterobacteriaceae* that harbor *mcr-1* and *bla*_NDM-5_ represent an urgent global health threat, currently lacking evidence-based therapeutic strategies. Furthermore, there are no novel antibacterial agents in the current drug development pipeline active against MCR-1- and NDM-producing *Enterobacteriaceae* (15). This highlights the critical need to optimize our existing antibiotics for the future occurrence of these pathogens in the clinical setting. In the present study, we determined that a triple combination of polymyxin B, aztreonam, and amikacin was able to eradicate *E. coli* MCR1_NJ, the first reported isolate in the United States to produce MCR-1 and NDM, at both low and high bacterial densities in an *in vitro* HFIM. This finding supports the idea that polymyxin B may still be an integral component of treatment in combination with other antimicrobials for *mcr-1*-harboring organisms whose polymyxin MICs are typically around the susceptibility cutoff. Against MCR1_NJ, a polymyxin B front-loaded regimen alone resulted in regrowth at high and low initial inocula. However, despite extensive selection pressure by polymyxin B, MCR1_NJ did not acquire a chromosomal polymyxin resistance mutation, and the polymyxin B MIC remained remarkably stable at 4 mg/liter through 10 days. The stability of polymyxin resistance additionally suggests that the administration of polymyxin antibiotics to *mcr-1-*harboring organisms is unlikely to have a collateral impact to potentiate high levels of polymyxin resistance such as have been described for polymyxin-induced chromosomal mutations in *mgrB* or *pmrAB* (16, 17). The effect of polymyxin monotherapy on polymyxin-susceptible *Enterobacteriaceae*, where high levels of resistance almost always emerge, is in stark contrast to the stable, low-level polymyxin resistance observed in *mcr-1-*harboring organisms (18). This may also support the use of short-duration polymyxin B regimens that can generate concentrations above the MIC.

Amikacin monotherapy or a combination of polymyxin B and amikacin resulted in rapid proliferation of MCR1_NJ amikacin-resistant subpopulations and corresponding increases in the amikacin MICs. Although AAC(6′)-Ib has been reported to confer amikacin and tobramycin resistance (19), *E. coli* MCR1_NJ was initially susceptible to amikacin despite harboring the *aac(6′)-Ib-cr* resistance gene. A majority of NDM-producing organisms are resistant to each of the aminoglycosides; however, previous surveillance studies have shown that amikacin may retain its bactericidal activity more frequently than gentamicin or tobramycin (20, 21). Further investigation is required to determine the precise mechanism of amikacin resistance proliferation and to determine its clinical relevance. The regrowth of *E. coli* MCR1_NJ upon exposure to amikacin represents a potentially significant clinical limitation to aminoglycoside utilization in the absence of genomic screening and further supports the significant promise of the triple-antimicrobial combination reported here.

Unexpectedly, aztreonam monotherapy or a combination of polymyxin B and aztreonam at a high initial inoculum of *E. coli* MCR1_NJ caused undetectable bacterial counts despite visible turbidity in the HFIM cartridges for 240 h. Thus, we used scanning electron microscopy to visualize cells following exposure to these two aztreonam-containing regimens and observed long, filamentous cells. The turbidity of the samples from the HFIM cartridge, with an absence of bacterial growth on agar plates and the visualized filamentous cells, demonstrates the remarkable capacity for MCR1_NJ to persist in the face of prolonged, intensive antibiotic exposure and is ultimately suggestive of a long, filamentous, nonreplicating persister (NRP) phenotype (Fig. 2) (22). The *E. coli* MCR1_NJ NRPs, caused by aztreonam monotherapy or a combination of polymyxin B and aztreonam, could not be grown or quantified by typical colony-counting techniques on antibiotic-free media (Mueller-Hinton agar or broth, grown for up to 7 days), likely due to their inability to divide. Importantly, these filamentous MCR1_NJ populations were not accompanied by aztreonam resistance, as aztreonam-resistant colonies were not detected in PAPs. Gladys Hobby et al. (23) and Joseph Bigger (24) first described the bacterial-persister phenomenon in the 1940s in reference to subpopulations of bacteria that were remarkably tolerant to antibiotics following exposure to penicillin. Subsequent studies regarding NRPs have revealed that despite retaining antibiotic susceptibility, they enter into an antibiotic-tolerant, slowly growing or nonreplicating state that can later be reverted to the original phenotype under specific conditions (25). The elongated and filamentous *E. coli* cells that were observed following aztreonam exposure are likely a product of penicillin binding protein 3 (PBP3) inhibition, which has been shown to inhibit cell division by prevention of cellular septation, subsequently leading to filamentation (22). Thus, bactericidal doses of aztreonam against a high density of MCR1_NJ likely killed the susceptible fraction of the total population, leaving behind an aztreonam-tolerant subpopulation of *E. coli* that subsequently elongated due to PBP3 inhibition. Interestingly, persister levels have been shown to increase sharply during late exponential growth, reaching ~1% of the population by stationary phase, which may help to explain the detection of the NRP phenotype following aztreonam exposure only at the higher bacterial density (26).

Although the clinical implications of nonreplicating, persistent filamentous cells are yet to be completely defined, since these bacteria often remain metabolically active and are capable of awakening from their tolerant phase following the removal of antibiotic pressure, they may be responsible for some recurrent or relapsing infections (27). These findings highlight potential limitations to certain aztreonam-based regimens against infections involving high bacterial densities of *mcr-1-* and *bla*_NDM-5_-coharboring *E. coli* strains that have been defined as aztreonam susceptible only by traditional MIC-testing techniques. However, aztreonam monotherapy may still be an important treatment strategy to combat low-bacterial-density infections caused by these pathogens, since no NRPs were observed at the low inoculum in the HFIM. Further studies are required to analyze the triple combination of polymyxin B, aztreonam, and amikacin against *E. coli* isolates that are aztreonam resistant, such as through the presence of *bla*_CTX-M_, while still coharboring *mcr-1* and *bla*_NDM-5_.

In summary, we have characterized for the first time the pharmacodynamics of a triple combination against *Enterobacteriaceae* coharboring mobile polymyxin and carbapenem resistance genes. The combination of polymyxin B, aztreonam, and amikacin may, through suppression of resistance and prevention of long, filamentous NRP formation, be an effective treatment option to combat infections caused by bacteria that harbor both *mcr-1* and *bla*_NDM-5_. The absence of a chromosomal mutation conferring additional polymyxin resistance in MCR1_NJ despite polymyxin B exposure could additionally support the use of this polymyxin-based combination strategy to treat infections with *mcr-1*-harboring organisms in an era of few treatment options. Additional studies should be conducted using animal models to fully elucidate the efficacy of this triple combination and the clinical relevance of NRPs prior to clinical evaluation and implementation.

### Methods.

*E. coli* isolate MCR1_NJ grown on Mueller-Hinton II agar (MHA) containing 2 mg/liter polymyxin B (Becton, Dickinson and Company, Franklin Lakes, NJ) was utilized for all experiments (4). The MICs for MCR1_NJ were obtained for all antimicrobials utilized in this experiment via broth microdilution in duplicate according to CLSI guidelines (Table 1) (28). Time-kill studies were conducted as previously described (29) at an inoculum of ~10^6^ CFU/ml to uncover the antimicrobial regimens with the greatest pharmacodynamic activity as defined by eradication at 24 h. The following antimicrobials that are commercially available in the United States for the treatment of infections caused by Gram-negative bacteria were tested alone or in combination with polymyxin B: amikacin (37.5 mg/liter), ampicillin-sulbactam (100/50 mg/liter), aztreonam (75 mg/liter), cefoxitin (65 mg/liter), ceftazidime (80 mg/liter), ceftazidime-avibactam (80/15 mg/liter), ceftolozane-tazobactam (55/15 mg/liter), chloramphenicol (32 mg/liter), ciprofloxacin (5 mg/liter), meropenem (50 mg/liter), nitrofurantoin (40 mg/liter), piperacillin-tazobactam (75/15 mg/liter), polymyxin B (6 mg/liter), rifampin (3.5 mg/liter), tigecycline (2 mg/liter), and trimethoprim-sulfamethoxazole (30/4 mg/liter). Antimicrobials that eradicated MCR1_NJ at 24 h in the time-kill experiments were selected for validation in the hollow-fiber infection model (HFIM). The two combinations that resulted in undetectable bacterial counts at 24 h were polymyxin B plus aztreonam and polymyxin B plus amikacin. To intensely investigate the concentration-response effect for these antibiotics alone and in the presence of polymyxin B, dose-ranging time-kill experiments were conducted at an inoculum of ~10^6^ CFU/ml of MCR1_NJ with polymyxin B (2.41 mg/liter) combined with either aztreonam (0.016, 0.063, 0.25, 1.0, 4.0, 16, 64, and 256 mg/liter) or amikacin (0.016, 0.063, 0.25, 1.0, 4.0, 16, 64, and 256 mg/liter).

**TABLE 1:** *E. coli* MCR1_NJ susceptibilities determined by broth microdilution for all antimicrobials utilized in the time-kill experiments and hollow-fiber infection model

Antimicrobial agent	MIC (μg/ml)
Amikacin	4
Ampicillin-sulbactam	>128/64
Aztreonam	≤0.25
Cefoxitin	>64
Ceftazidime	>64
Ceftazidime-avibactam	>16/4
Ceftolozane-tazobactam	>256/4
Chloramphenicol	>64
Ciprofloxacin	>64
Meropenem	>64
Nitrofurantoin	16
Piperacillin-tazobactam	128/4
Polymyxin B	4
Rifampin	>64
Tigecycline	0.50
Trimethoprim-sulfamethoxazole	2/38

The HFIM studies were conducted over 10 days using cellulosic cartridges (cartridge C3008; FiberCell Systems, Inc., Frederick, MD) with 18 ml of either ~10^6^ or ~10^8^ CFU/ml of *E. coli* MCR1_NJ, as described previously (30). Polymyxin B (half-life [*t*_1/2_] = 8 h), aztreonam (*t*_1/2_ = 2 h), and amikacin (*t*_1/2_ = 2 h) regimens were simulated alone or in double or triple combinations in the HFIM based on human pharmacokinetic data as follows (31–33): (i) polymyxin B front load of 3.33 mg/kg for 1 dose followed by 1.43 mg/kg every 12 h (q12h) starting 12 h later, with a maximum concentration of free, unbound drug (*fC*_max_) of 3.61 mg/liter and an area under the concentration-time curve from 0 to 24 h for the free, unbound fraction of drug (*f*AUC_0−24_) of 48.2 mg ⋅ h/liter during the first day and an *fC*_max_ of 2.41 mg/liter and *f*AUC_24_ of 35.9 mg ⋅ h/liter at steady state; (ii) aztreonam at 2 g q8h with an *fC*_max_ of 95.3 mg/liter and *f*AUC_0−24_ of 807.9 mg ⋅ h/liter during the first day and an *fC*_max_ of 95.3 mg/liter and *f*AUC_24_ of 827.2 mg ⋅ h/liter at steady state; and (iii) amikacin at 15 mg/kg q24h with an *fC*_max_ of 50.1 mg/liter and *f*AUC_24_ of 153.6 mg ⋅ h/liter throughout every day.

The MCR1_NJ baseline MICs for polymyxin B, aztreonam, and amikacin were 4, ≤0.25, and 4 mg/liter, respectively (Table 1). For combination regimens, polymyxin B was supplemented into the central reservoir of the HFIM every 1.2 h to maintain a pharmacokinetic profile consistent with a *t*_1/2_ of 8 h (34). Total bacterial counts and population analysis profiles (PAPs) were performed during the HFIM by plating 50-μl aliquots of bacterial samples onto MHA plates without drug for total counts or onto plates containing polymyxin B (2, 4, 8, 16, and 32 mg/liter), aztreonam (0.25, 2, and 16 mg/liter), or amikacin (4, 16, or 64 mg/liter) for PAPs. The antibiotic concentrations in the HFIM were validated using samples that were obtained over a 48-h period. Polymyxin B concentrations were determined by a liquid chromatography (LC) single-quadrupole mass spectrometry (MS) method described previously (35). Amikacin concentrations were validated using a liquid chromatography-tandem mass spectrometry (LC-MS/MS) method (Dionex ultra-high-performance liquid chromatography [UHPLC] and triple-stage quadrupole [TSQ] Endura instruments; Thermo Scientific, Waltham, MA). The amikacin calibration curve from 1 to 75 mg/liter was linear, with an overall assay precision ranging from 1.7 to 4.9%. Aztreonam concentrations were analyzed using an LC-MS/MS method (Agilent series 6460 triple-quadrupole LC-MS/MS coupled with Agilent series 1260 LC; Agilent Technologies, Santa Clara, CA). The compound was extracted by protein precipitation using acetonitrile containing amdinocillin as an internal standard. A gradient method was employed, with the mobile phase consisting of water with 0.1% formic acid and acetonitrile with 0.1% formic acid. The calibration curve was linear over a range of 1 to 100 mg/liter (*R*^2^ = 0.997). There was satisfactory agreement between the observed and targeted pharmacokinetic profiles for polymyxin B (*R*^2^ = 0.93, slope = 0.69, and intercept = −0.02), amikacin (*R*^2^ = 0.99, slope = 0.87, and intercept = 1.90), and aztreonam (*R*^2^ = 0.98, slope = 0.86, and intercept = 9.05).

Whole-genome DNA sequencing (WGS) and analyses were performed as previously described (36) to assess the influence of polymyxin B exposure on the *E. coli* MCR1_NJ genotype. The sequences of *E. coli* MCR1_NJ prior to antibiotic exposure (0 h), the *E. coli* MCR1_NJ unexposed growth control (240 h), and *E. coli* MCR1_NJ after polymyxin B exposure (24 h and 240 h) were compared. Samples for sequencing were obtained from the HFIM at the respective time points, and genomic DNA was extracted using a Wizard Genomic DNA purification kit (Promega, Madison, WI). The Nextera DNA library preparation kit was used for genome library preparation, and the library was then sequenced using the Illumina NextSeq platform (Illumina, San Diego, CA). Quality-trimmed reads of mutants were aligned to the reference genome (MCR1_NJ, accession number MAJK00000000) using the Burrows-Wheeler Aligner (37). Single-nucleotide polymorphisms (SNPs) and insertion-deletion sites (indels) were called using SAMtools (38) and VarScan (39), followed by annotation using snpEff (40). The default setting of >75% for variant allele frequency was used to call homozygous variants (SNPs and indels), and allele frequencies of between 10 and 75% were used to detect heterozygous variants.

Scanning electron microscopy (SEM) using a field emission scanning electron microscope with an Oxford energy-dispersive X-ray spectrometer (Hitachi SU70; Hitachi High Technologies America, Inc., Schaumburg, IL) was utilized to visualize *E. coli* MCR1_NJ. Images were obtained from HFIM samples prior to antibiotic exposure (0 h) and from 2 experimental samples (24 h and 240 h) where turbidity in the cellulosic HFIM cartridges was observed without cell growth on MHA plates (aztreonam monotherapy and polymyxin B plus aztreonam against the ~10^8^-CFU/ml *E. coli* MCR1_NJ starting inoculum).

### Accession number(s).

The sequence reads of this study have been deposited under BioProject accession number PRJNA353361 with accession numbers SRX2772346 to SRX2772348.
